# Flexible Sensors for Pressure Therapy: Effect of Substrate Curvature and Stiffness on Sensor Performance

**DOI:** 10.3390/s17102399

**Published:** 2017-10-20

**Authors:** Iryna Khodasevych, Suresh Parmar, Olga Troynikov

**Affiliations:** School of Fashion and Textiles, Royal Melbourne Institute of Technology, Melbourne 3056, Australia; iryna.khodasevych@rmit.edu.au (I.K.); s3524484@student.rmit.edu.au (S.P.)

**Keywords:** pressure sensor, sensor evaluation, calibration, curvature, stiffness, accuracy, drift, piezoresistive sensors

## Abstract

Flexible pressure sensors are increasingly being used in medical and non-medical applications, and particularly in innovative health monitoring. Their efficacy in medical applications such as compression therapy depends on the accuracy and repeatability of their output, which in turn depend on factors such as sensor type, shape, pressure range, and conformability of the sensor to the body surface. Numerous researchers have examined the effects of sensor type and shape, but little information is available on the effect of human body parameters such as support surfaces’ curvature and the stiffness of soft tissues on pressure sensing performance. We investigated the effects of body parameters on the performance of pressure sensors using a custom-made human-leg-like test setup. Pressure sensing parameters such as accuracy, drift and repeatability were determined in both static (eight hours continuous pressure) and dynamic (10 cycles of pressure application of 30 s duration) testing conditions. The testing was performed with a focus on compression therapy application for venous leg ulcer treatments, and was conducted in a low-pressure range of 20–70 mmHg. Commercially available sensors manufactured by Peratech and Sensitronics were used under various loading conditions to determine the influence of stiffness and curvature. Flat rigid, flat soft silicone and three cylindrical silicone surfaces of radii of curvature of 3.5 cm, 5.5 cm and 6.5 cm were used as substrates under the sensors. The Peratech sensor averaged 94% accuracy for both static and dynamic measurements on all substrates; the Sensitronics sensor averaged 88% accuracy. The Peratech sensor displayed moderate variations and the Sensitronics sensor large variations in output pressure readings depending on the underlying test surface, both of which were reduced markedly by individual pressure calibration for surface type. Sensor choice and need for calibration to surface type are important considerations for their application in healthcare monitoring.

## 1. Introduction

Over the past decade, digital health monitoring systems have developed rapidly in response to greater health awareness and rising health care costs [1,2,3,4]. Among these systems, pressure sensing has attracted considerable attention due to its potential in products spanning consumer, industrial, and biomedical applications [5,6,7,8,9]. Of particular note is its application in compression therapy, which is a major treatment and/or support for treatments in phlebological diseases such as chronic venous insufficiency, varicose veins and venous leg ulcers [10,11]. Compression therapy involves use of elastic and inelastic medical pressure garments, bandages and external intermittent compression therapy; it can be used over the short or long term and as a maintenance therapy. Numerous standards apply to medical pressure garments with respect to sizing, materials and the induced pressure gradients to the wearers’ limbs. An interface pressure range and a pressure at specific sites of the limb—such as calf to ankle and thigh to ankle—are defined for each graduated compression garment. The appropriate size of the garment is also defined with respect to specified body measurements such as leg circumference at ankle, calf and thigh. There are many possible variations in these parameters; digital pressure sensors offer medical personnel a simple means of determining whether the compression garment is delivering the desired pressure to the patient.

Previous researchers have used commercially available flexible piezoresistive force sensors for determination of pressures, such as in-shoe pressure, to aid diagnosis of limb problems and prevent injury [12,13]. These devices are also widely used to determine interface pressure during compression-therapy-based treatment of chronic venous disorders [14,15,16] where interface pressure reflects the perpendicular pressure exerted by a pressure-inducing garment or bandage on an underlying limb. However, the accuracy and repeatability of piezoresistive sensors are dependent on their polymer substrate, and, owing to the viscoelastic nature of the polymer, these sensors often show drift variations [17,18]. It is imperative to verify the sensing performance and suitability of these types of pressure sensors for a given application in conditions as close to the practical application as possible. These conditions include the curvature of the surface at the site where the measurement takes place, as well as the stiffness of that surface.

Flexible piezoresistive force sensors (or force-sensing resistors—FSRs) are mostly used as pressure sensors. They are constructed of a force-sensing piezoresistive material with electrical resistance properties that vary with an applied pressure, enabling calculations of force per unit area. Generally, as the pressure is increased, the resistance of these piezoresistive materials falls due to percolation or quantum tunnelling effects that result in incremental increases in the number of conductive paths [19]. Researchers have used commercial FSRs such as Interlink FSR (Interlink Electronics Inc., Camarillo, CA, USA) and Tekscan Flexiforce^®^ (Tekscan Inc., South Boston, MA, USA) to determine interface pressures [18,20,21], where the sensors are calibrated on a rigid flat surface. However, unlike the flat and relatively rigid surfaces present in applications such as shoe insoles, many human body surfaces vary in geometry, curvature and stiffness. In the studies mentioned above, the surfaces and methods used for calibration of the pressure sensors were different from those used in loading tests. In addition, these studies did not employ realistic human-leg-like test conditions; therefore, the accuracy, repeatability of the sensor output, and as a result general suitability of the sensors are uncertain.

Only a few researchers have studied the effect of pressure application surface attributes such as curvature, geometry and stiffness on the sensing performance of flexible pressure sensors. Ferguson-Pell et al. [22] found that change in curvature affected both the offset value and sensitivity of the sensor. However, their study was restricted to rigid cylinders with radii of curvature ranging from 8.0 mm to 51.7 mm, some of which are not comparable to the measurements of human limbs such as arms and legs. In addition, their study involved only a single sensor (Tekscan Flexiforce^®^), and was published in 2000, well before several other thin film pressure sensors became available. Moreover, this study did not include dynamic testing, being conducted in a static condition using dead weights. Komi et al. [23] also studied the effect of surface curvature on the output of sensors for golfing applications, and confirmed that the change in surface curvature of the sensor led to an increase in error. However, this study involved high pressures, corresponding to up to 111 N of force, beyond those encountered in most medical pressure applications. Recently, Likitlersuang et al. [24] studied a Flexiforce sensor at the body/device interface. They found that with conventional techniques of sensor calibration and application the errors were in the range of 23.3 ± 17.6%, reduced to 2.9 ± 2.0% on application of a thin rigid disc between the sensor and human body and calibrating using a compliant surface. However, they did not assess the effect of dynamic testing conditions on human-body-like soft tissue; instead, their testing was performed on a volunteer’s forearm, limiting the repeatability of the tests.

The present paper describes a study that formed part of an extensive experimental research program, conducted by our group at RMIT University (Royal Melbourne Institute of Technology), to design and evaluate body-conformable pressure sensors for pressure therapy applications. We investigated the effect of changes in underlying surfaces on the pressure sensing performance of two commercially available sensors on a human-leg-like test setup that is representative of human soft tissue surfaces. The sensors were selected from a wide range of commercial sensors based on our previous research [25], which demonstrated that they were the most suitable for compression therapy. The pressure measurements were conducted in a low pressure range (20–70 mmHg) appropriate for compression therapy treatment. We evaluated the effect of surface curvature and the stiffness of the underlying surface on sensing performance parameters such as accuracy, drift and repeatability. We also considered the different methods of calibration of the sensors. Loading tests were performed to gain a better understanding of possible influences of stiffness and curvature during both static and dynamic conditions of practical pressure garment use.

## 2. Materials and Methods

The experimental methods employed in this study included reference test surfaces whose parameters, such as radius of curvature and stiffness simulated those of the human leg at the ankle, calf and thigh. The two sensors, manufactured by Peratech Holdco Ltd. (Richmond, UK) and Sensitronics LLC (Bow, WA, USA), were used to evaluate the influence of the stiffness and curvature of these surfaces on accuracy and repeatability of pressure measurements in both static and dynamic testing environments.

### 2.1. Sensors Used for Evaluation

**Figure 1:**
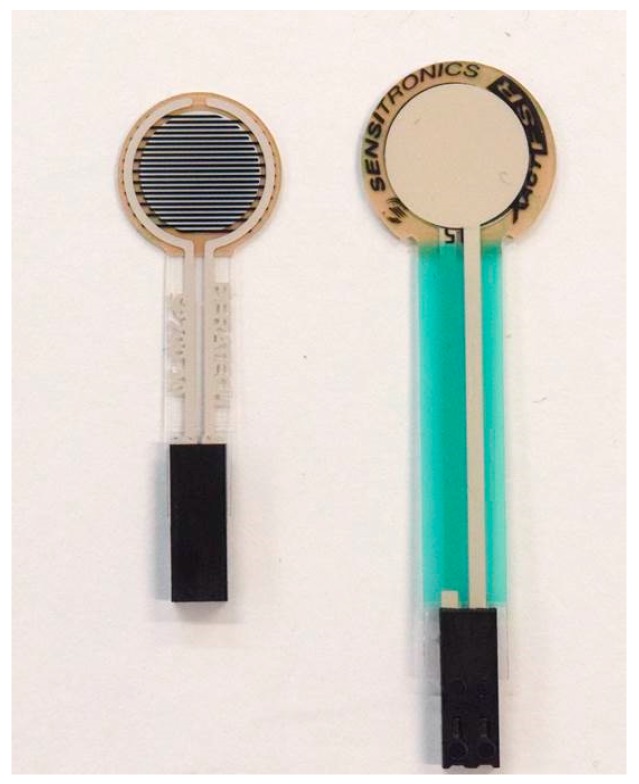
Piezoresistive force sensors used for evaluation: (**left**) Peratech QTC™; (**right**) Sensitronics^®^ FSR.

This study was performed using two low-cost commercially available thin and flexible piezoresistive force sensors: the Peratech quantum tunnelling composite (QTC)^TM^ SP 200-10 sensor and the Sensitronics^®^ Half Inch ThruMode FSR (Figure 1). These sensors were selected on the basis of a previous study [25] that determined the most suitable and the best-performing of five pressure sensors suitable for compression therapy applications.

Both of these sensors are piezoresistive sensors with high resistance in an unloaded state. When pressure is applied, an increase in the number of conductive paths in the sensing material causes a reduction in the electrical resistance offered by the sensing material (piezoresistive conductive ink applied between printed electrical contacts and polymer sheets). The geometry and the sensing properties of the sensors evaluated in this study are presented in Table 1.

### 2.2. Experimental Setup

**Figure 2:**
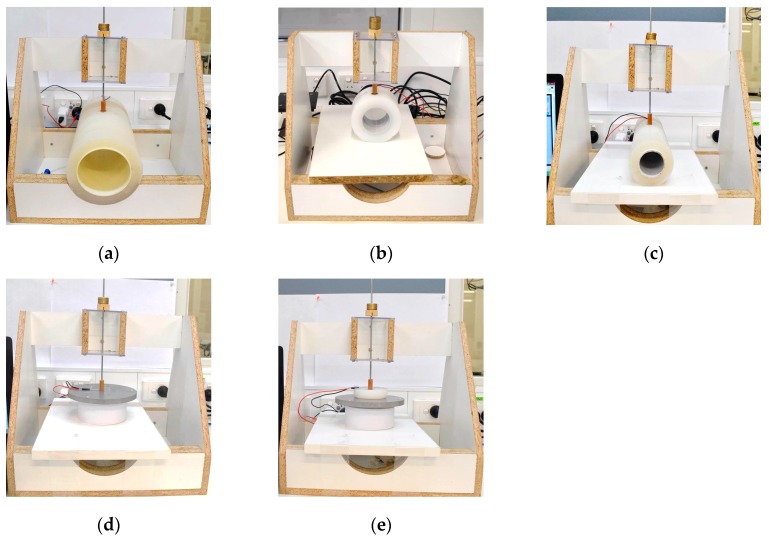
Experimental setup showing test surfaces: (**a**) test cylinder with 13 cm diameter; (**b**) test cylinder with 11 cm diameter; (**c**) test cylinder with 7 cm diameter; (**d**) rigid flat aluminium surface; (**e**) soft flat surface with 2 cm thick silicone layer.

A custom-made wooden test apparatus [25] was used to simulate the actual pressure measurement conditions on a human leg in compression therapy (Figure 2). This test apparatus enabled accurate placement of cylinders and flat plates to attain consistent results. The effect of surface curvature was studied using three cylinders with diameters 13 cm, 11 cm and 7 cm, mimicking the size of an average adult male leg at calf, midpoint between calf and ankle, and at ankle [28], respectively (Figure 2a–c). The cylinders were constructed from polyvinyl chloride pipes with external diameters of 9 cm, 7 cm, and 4.4 cm, respectively, over which we applied room-temperature-vulcanised animatronic-skin-grade silicone with hardness of 10 Shore A (sourced from Dalchem Chemicals, Melbourne, Australia). Tests on a flat rigid surface were performed on an aluminium circular plate with diameter 13 cm (Figure 2d). A 2 cm thick sheet of the same animatronic-grade silicone with a diameter of 7 cm was placed on this surface to simulate a flat soft surface (Figure 2e).

The sensors were affixed at marked spots on the top of the surfaces, using transparent adhesive tape only on the electrodes of the sensors to avoid additional load. The pressure was applied by a t-pin with a flat bottom diameter of 9.4 mm, which had a circular platform on its top to hold standard dead weights. The pressure exerted by the dead weights on the sensor was converted to mmHg beforehand and ranged from 17.63 to 94.03 mmHg.

### 2.3. Data Acquisition System

The data was acquired by RMIT University’s proprietary National Instrument-based electronic setup, run via LabView (version 2013, National Instruments Corporation, Austin, TX, USA). The custom-designed software interpreted this acquired raw data in real time and calculated the pressure-voltage relationship.

### 2.4. Test Procedure

The calibration and load application was carried out using dead weights on the t-pin platform [25]. The weights on sensors were increased in increments of approximately 10 mmHg. Both sensors were subjected to a series of static and dynamic tests on all five surfaces and the corresponding applied pressure parameters were derived and evaluated. Standard room temperature of 20 °C and relative humidity of 65% RH were maintained throughout the tests.

● Sensor Calibration

Dead weights were added onto the sensor starting from 10 mmHg and finishing with 90 mmHg in a stepped incremental pattern with 30-s intervals. Prior to applying each increment of pressure through the addition of the next weight, the previous weight was removed and the sensor was allowed to return to zero pressure state to avoid a continuous drift effect. The calibration procedure was repeated five times to obtain five output voltage vs applied pressure curves.

Calibration on the flat rigid surface (Figure 2d) was performed for both sensors. The five sets of output voltage vs applied pressure data were exported to Matlab (version 9.1, The Mathworks, Inc., Natick, MA, USA) and coefficients of linear fit were found. The output pressure readings were then obtained from measured output voltages as

(1) P=a⋅V+b

where a and b are the coefficients of the fit. To test the possibility of using the same calibration for other test surfaces, this flat rigid surface calibration was used to obtain output pressure readings from measured output voltages in all tests.

**Figure 3:**
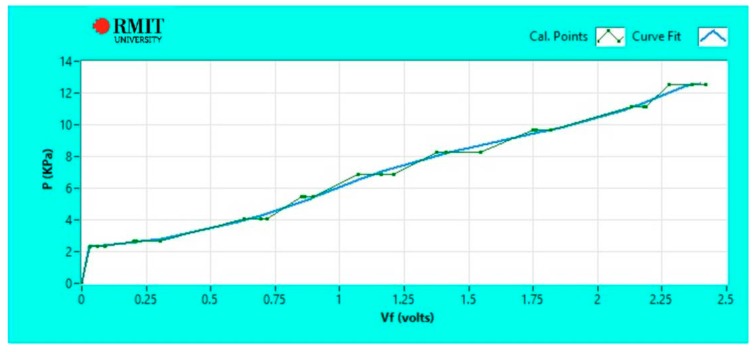
Calibration curve in LabView denoting the pressure-voltage relationship for the Sensitronics sensor.

In addition to the approach detailed above, the calibration was also performed individually on each type of surface for both Peratech and Sensitronics sensors. Again, five sets of measurements for output voltage vs. applied pressure were obtained for each type of surface while adding weights onto the sensor, following the same procedure as described above. These datasets for each surface were than fitted using LabView's inbuilt B-Spline fit as shown in Figure 3. B-Spline fit follows measured data more closely than linear fit, but if the number of repeats is increased, the B-Spline fit should asymptotically approach linear fit. These fitted curves were used as calibration data files for each surface in order to derive output pressure readings from measured output voltage. The output pressures were determined by LabView software and displayed on the screen during the tests.

● Load Application

The reference values stored for each sensor in the calibration data files were recalled in order to determine parameters such as output pressure accuracy and drift behaviour in response to applied pressure. Static and dynamic load applications were performed in a systematic manner to determine the effect of surface curvature and stiffness on the sensors' pressure sensing performance. A pressure range of 20–70 mmHg is required for compression therapy, so it was decided to select three pressure points in this range: 30 mmHg, 50 mmHg and 70 mmHg. The nearest pressure points in our dataset—30.5 mmHg, 51.4 mmHg and 72.7 mmHg—were used for loading tests and evaluation of the sensors.

Static evaluation was performed by application of a static load for eight hours, creating conditions identical to prolonged wear of a compression garment under a constant pressure. Dynamic testing was performed by applying 10 cyclic loads at 30 s intervals in a duty cycle of 50% (period = 60 s); this simulated short-term changes in pressure during bandage application and physical activity.

● Sensing parameter and data analysis

The raw sensor output in terms of voltage signal was subsequently processed to derive sensing parameters [25] such as output pressure reading accuracy, voltage and pressure drift behaviour and repeatability of pressure readings at high, medium and low applied pressures.

We assessed sensor accuracy under both static and dynamic conditions, calculating it as the percentage agreement between measured and applied pressure. We defined drift as the change in output signal independent of the applied pressure, and calculated it as a percentage of the voltage or pressure measured when the rate of change of the output had reduced sufficiently to be considered steady state. The detailed approach is described in [25]. We determined the repeatability of the sensor output by comparing the average accuracy values and deviation of parameter values from average accuracy over 10 cycles of dynamic tests.

## 3. Results

### 3.1. Static Evaluation

**Figure 4:**
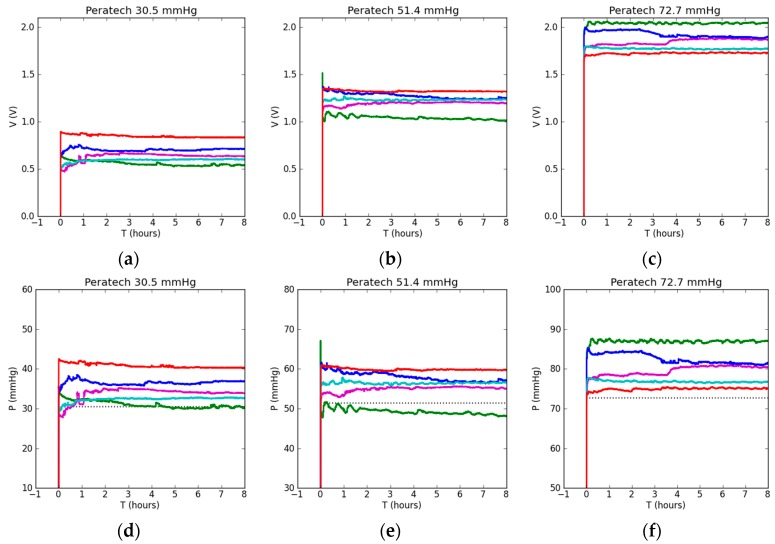

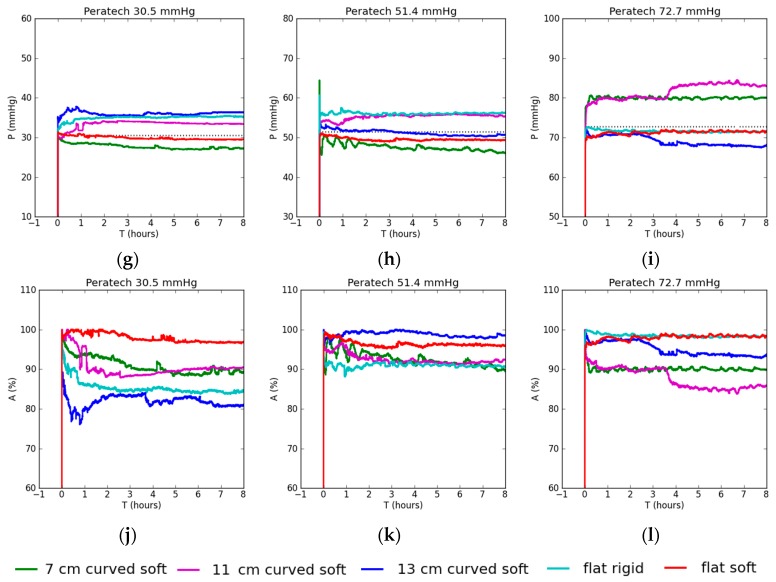
Static testing results using Peratech sensor with applied pressure shown in the title: (**a**–**c**) sensor output voltage reading; (**d**–**f**) sensor pressure reading when flat rigid calibration was used for all tests; (**g**–**i**) sensor pressure reading when sensor was recalibrated before each test. Dotted lines show corresponding applied ‘gold standard’ pressures; (**j**–**l**) accuracy of the sensor pressure reading.

Experimental results from eight hours of static testing using the Peratech sensor are shown in Figure 4. Figure 4a–c show output voltages for 30.5, 51.4 and 72.7 mmHg of applied pressure respectively. Voltage response characterises the intrinsic behaviour of the sensor before any post-processing and was not affected by the choice of calibration. For 30.5 and 51.4 mmHg pressures, there were increases in output voltage for the same applied pressure in the following sequence: 7 cm curved soft surface, flat rigid surface, 11 cm curved soft surface, 13 cm curved soft surface and finally flat soft surface achieving the highest voltage. Generally, as the radius of curvature increased, so did the output voltage, but there was some overlapping. For 72.7 mmHg of applied pressure, the flat soft case produced the lowest output voltage and the 7 cm curved surface produced the highest, reversing the results achieved at lower pressures; outputs for the flat rigid, 11 cm and 13 cm curved surfaces followed the same order of voltage increase as for lower pressure values. The sensor reached its final output value very quickly and then showed very stable behaviour in all cases, with negligible drift in output voltage and little deviation from average. The difference between the lowest and the highest output voltages was about 0.4 V at all pressures. However, because the average output voltage increased with pressure, the deviation from the average output voltage produced on all different surfaces progressively diminished from around 50% at lower pressures to 30% and then to 20% for higher applied pressures. A large difference in voltage can be seen between soft and rigid flat surfaces at lower applied pressure, which reduces to nearly the same voltage as pressure is increased.

Figure 4d–f show sensor output pressure results after a linear calibration fit for the flat rigid surface (parameters of which were calculated in Matlab) was applied and output voltage values were correlated to corresponding pressure values via post-processing. This emulated the sensor being calibrated on a flat rigid surface, for example at the factory or testing laboratory, and then used in a practical application on soft curved human limb without further adjustment. Due to the linear correlation between output voltage and pressure applied to the sensor, linear fit was a good physical choice. Pressure values extracted via this method follow the output voltage trends closely. Differences of 10–15 mmHg between lowest and highest pressure values were recorded for different surfaces.

Further tests were conducted and each surface setup was individually calibrated and fitted using inbuilt LabView B-Spline fit. The aim was to minimize the differences between various types of surfaces via individual calibration, so there was no particular order for the pressure curves to follow; that is, a particular voltage does not necessarily correspond to the same pressure value across all test setups after applying the calibration. Ideally, all the curves would overlap and report the same calculated pressure equal to the applied pressure. The results of the measurements are presented in Figure 4g–i. We recorded the least difference between lowest and highest pressure output readings across different setups at 51.4 mmHg of applied pressure. The flat soft surface case demonstrated exceptionally high correspondence to actual applied pressure at all pressure levels. At 72.7 mmHg, high overlap was achieved in pressure readings for the flat soft, flat rigid and 13 cm curved soft setups, as well as in control pressure. Higher pressure was recorded for the 7 and 11 cm curved soft surface cases, which could be explained by the high curvature of the setup affecting pressure application. There was a slight reduction in the pressure difference between setups, however, it can be concluded that this sensor already operated at the maximum of its capability and individual calibration did not have a significant effect. The observed differences in output pressure readings can be attributed to the repeatability limitations of the sensor and not to the types of underlying surfaces.

Accuracies of pressure measurements achieved after individual calibrations for each surface type are shown in Figure 4j–l. Medium and high-pressure readings are highly accurate, exceeding 90%, except in the case of the 13 cm curved surface at 30.5 mmHg. For the flat soft and 13 cm curved cases, accuracies of 95.6–98.2% were achieved at medium and high pressures. The flat rigid case was the most accurate, at 98.7% at 72.7 mmHg. The 11 cm and 7 cm surfaces' measurements were around 90% accurate. Low pressure readings were least accurate, dropping to nearly 80%. The results are summarised in Table 2.

**Figure 5:**
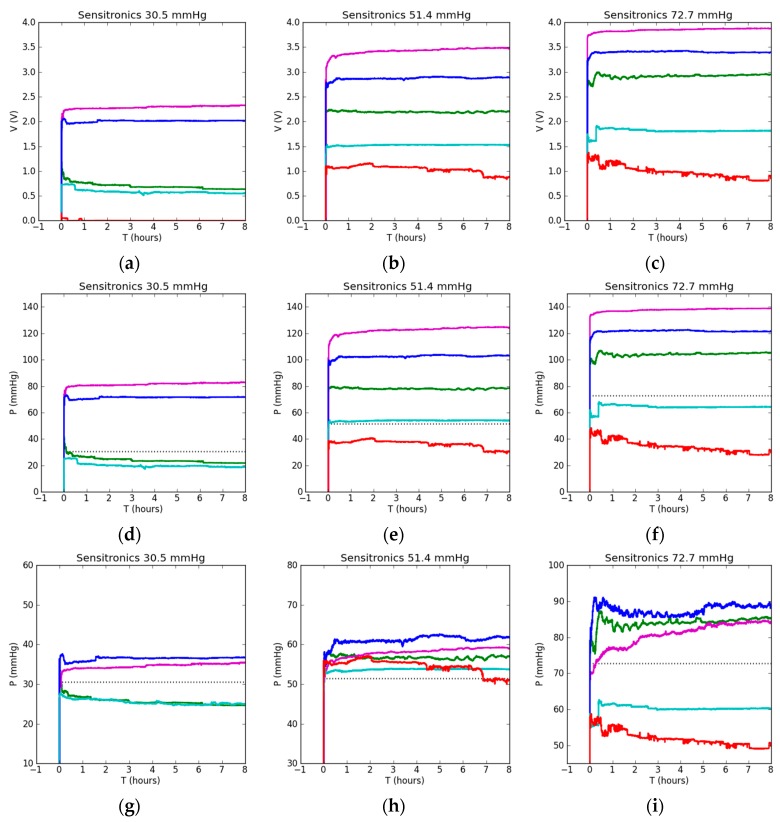

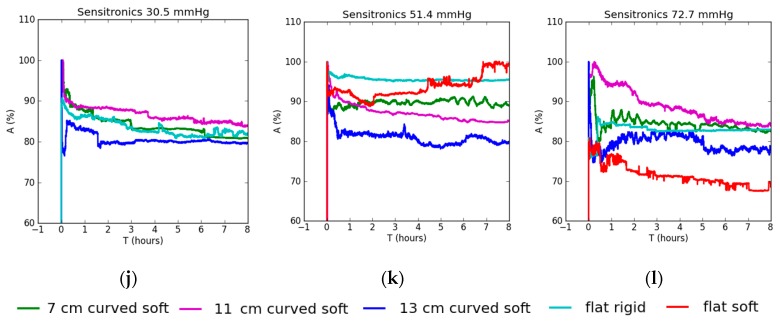
Static testing results for the Sensitronics sensor with applied pressure shown in the title: (**a**–**c**) sensor output voltage reading; (**d**–**f**) sensor pressure reading when flat rigid calibration is used for all tests; (**g**–**i**) sensor pressure reading when sensor is recalibrated before each test. Dotted lines show corresponding applied ‘gold standard’ pressures; (**j**–**l**) accuracy of the sensor pressure reading.

The Sensitronics sensor produced significantly larger differences in output parameters than the Peratech sensor, depending on the test setup (Figure 5). As can be seen from Figure 5a–c, the output voltage difference was up to 4 V between the lowest and highest values and this difference increased with applied pressure. The order of voltage increase in relation to test surface was consistent across all pressures. The flat soft setup resulted in the lowest output voltage, followed by the flat rigid, 7 cm curved soft, 13 and 11 cm curved soft surface. Curved surfaces resulted in consistently higher voltage output. The flat soft setup output voltage for 30.5 mmHg of applied pressure was near zero, so pressure could not be determined. This sensor takes longer to reach its steady state output value, with variations visible shortly after pressure application, but, once in steady state, it shows stable behaviour in all cases (except the soft flat surface), with little drift in output voltage.

After applying linear calibration fit for flat rigid surface, pressures were calculated from measured voltages, as shown in Figure 5d–f. Without individual calibrations for a surface type, pressure readings can be twice the actual applied pressure. Curved surfaces mostly result in overestimated pressure, but the pattern does not follow the radius of curvature, with 7 cm resulting in lowest pressure reading and 11 cm the highest. The soft flat surface resulted in significant underestimation of pressure.

Again, in order to address significant differences in output pressure readings obtained using calibration on a single surface only, individual calibrations were applied when measuring each surface type; its corresponding pressure readings are shown in Figure 5g–i. The closest readings to applied pressure were achieved at 51.4 mmHg of pressure, with both flat setups showing the closest result to actual applied pressure. For 72.7 mmHg, the difference between the flat and curved case results was still large but reduced compared to flat rigid calibration. All of the curved setups showed less deviation in output pressure readings between them.

Accuracy plots in Figure 5j–l show higher accuracy shortly after pressure application and subsequent drift towards lower values for all cases. This is related to calibration timing (30 s in this study) and corresponding choice of initial output value from which the drift is calculated. As a result of this investigation, the calibration timing for static measurements should be extended until the sensor output fully stabilizes. This timing is different for different sensors, but, in a static case, can be from a few minutes to an hour long. Most of the readings achieved below 90% accuracy, with exception of both flat cases at 51.4 mmHg and the 7 cm curved case at 72.7 mmHg, achieving 90–96.4% accuracy. Full results are shown in Table 2. The Sensitronics sensor benefits greatly from applications of individual setup calibration as it is more sensitive to the underlying surface.

**Figure 6:**
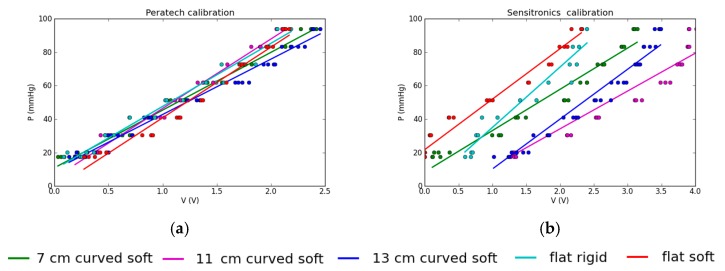
Measured calibration curves for (**a**), Peratech; (**b**) Sensitronics. Calibration points shown as dots. Linear fits for each type of surface shown as lines.

Figure 6 compares the two sensors’ calibrations for output voltage vs. applied pressure, obtained by linear fits in Matlab. For Peratech, the calibration curves are close together and overlap, confirming that the type of surface has minimal effect on the sensor performance. Sensitronics, on the other hand, has widely spaced calibration curves. Top lines are for the flat surfaces and lower lines correspond to curved surfaces. Deviations of actual measured values (dots) from a straight line on the calibration curve (fit) result in deviations of pressure readings from the average, which is visible in pressure plots. Thus, this sensor is more sensitive to the underlying surface. Behaviour in terms of amount of deviation from the linear fit is similar for all surfaces for each sensor.

**Figure 7:**
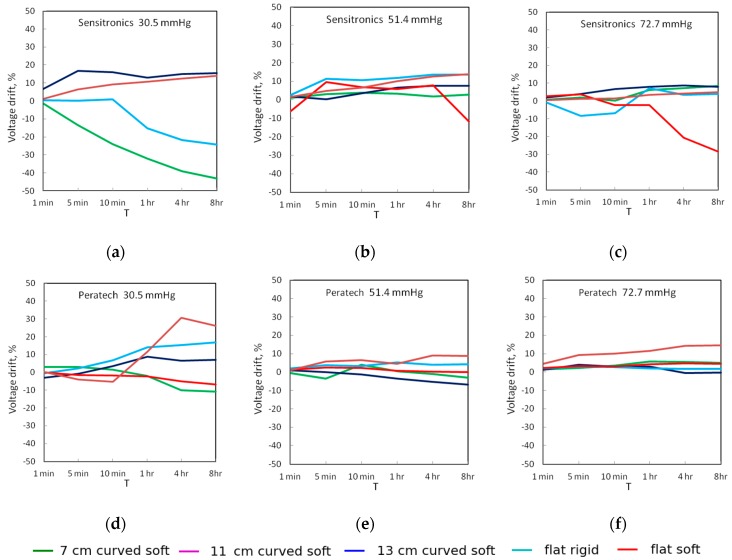
Static drift of output voltage for sensors at the three applied pressures: (**a**–**c**) Sensitronics; (**d**–**f**) Peratech.

**Figure 8:**
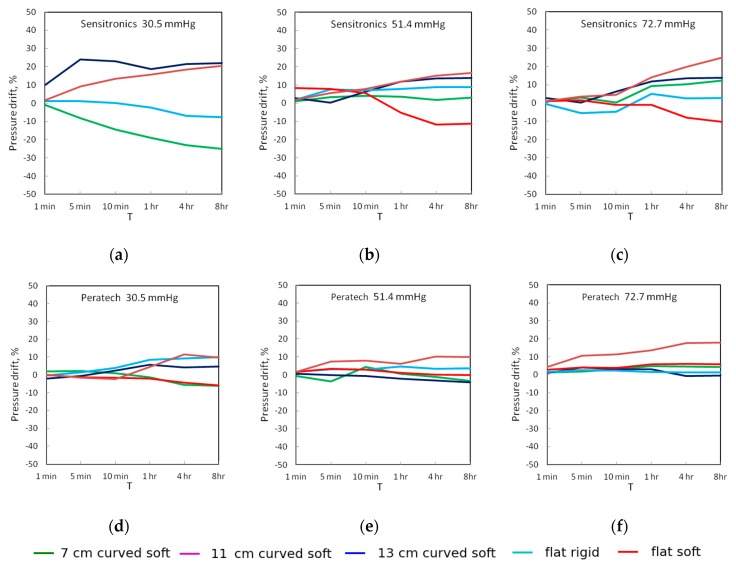
Static drift of measured pressure for sensors at the three applied pressures: (**a**–**c**) Sensitronics; (**d**–**f**) Peratech.

Figure 7 and Figure 8 show the drift in the output voltage and measured pressures as percentages of initial value measured 26 s after the application of pressure for up to eight hours. For the Peratech sensor, 8-h drift reduced with increased applied pressure across all test setups, starting from 6.7–16.8% for voltage with an outlier of 26% and 4.6–10% for pressure at 30.5 mmHg, 0–8.7% for voltage and 0–4% for pressure at 51.4 mmHg, and 0.3–5% for voltage and 0.4–6% for pressure at 72.7 mmHg with an outlier at 14%. The 11cm and 13 cm setups showed the largest voltage drift, while flat setups had the smallest drift for medium and high applied pressures. At low applied pressures flat setups drifted more and were comparable to curved setups.

For the Sensitronics sensor, after eight hours at 30.5 mmHg, drift was 15–40% for voltage and 7.5–25% for pressure. At 51.4 mmHg, drift was 2.8–13.5% for voltage and 3–16% for pressure. At 72.7 mmHg, drift was 2.9–13.5% for voltage and 2.6–24% for pressure. These data show that the Peratech sensor displayed more stable operation than the Sensitronics sensor. Both upward and downward drift was observed, with output pressure readings generally following the same trend as output voltage. The Sensitronics sensor displayed predominantly upward drift, especially for curved surfaces, with downward trends for the soft flat surface at medium and high applied pressures and flat rigid and 7 cm curved surfaces at low applied pressure. However, no definite trend could be assigned for a particular test setup since the direction of drift changed for different pressure levels. Drift changing direction might be attributable to the changing environment and internal structure within the sensor.

### 3.2. Dynamic Evaluation

**Figure 9:**
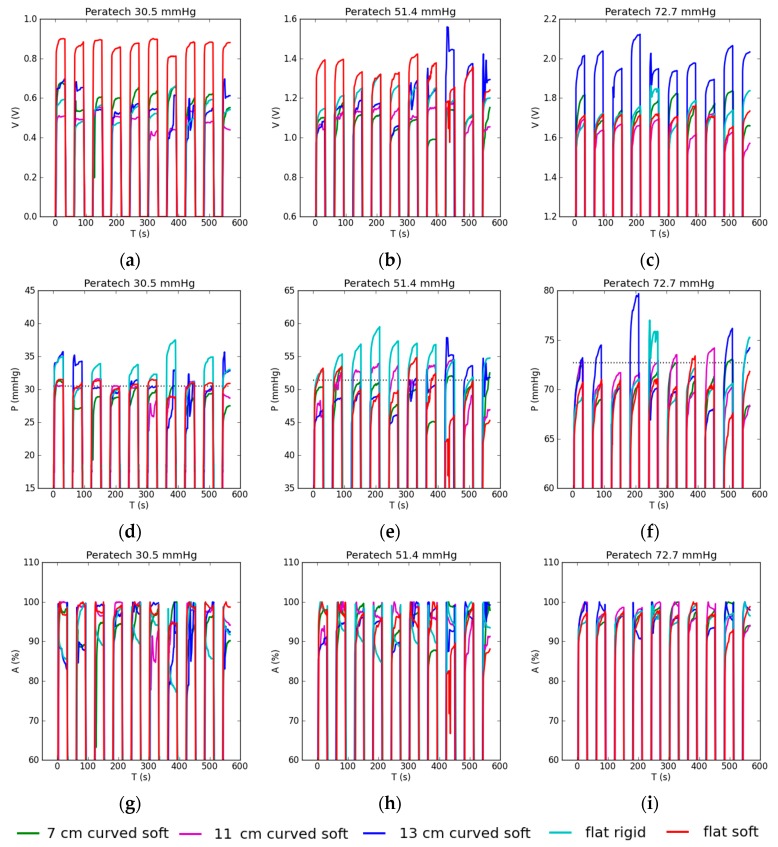
Dynamic testing results for Peratech sensor with applied pressure shown in the title: (**a**–**c**) sensor output voltage reading; (**d**–**f**) sensor pressure reading when sensor is recalibrated before each test. Dotted lines show corresponding applied gold standard pressures; (**g**–**i**) accuracy of the sensor pressure reading.

Dynamic testing results using the Peratech sensor are shown in Figure 9. As seen in Figure 9a–c, output voltage results overlapped, so the sensor has low sensitivity to the type of surface underneath it and better performance than the static case. At 30.5 and 51.4 mmHg, the flat soft case stands out as having consistently higher voltage results. At 72.7 mmHg, the 13 cm curved soft case displayed higher voltage than other setups.

After applying individual pressure calibrations for each type of surface, even more consistent results for output pressure were achieved across all Peratech sensor test setups, with very few outliers and excellent correspondence to actual applied pressure (Figure 9d–f). This was a result of individual calibration procedures for each test surface working as intended. Some degree of drift is visible in all cycles. Drift is smooth and upward in value, with nearly no erratic behaviour.

Consistent pressure results are reflected in predominantly high accuracy of 95–97.9% for most test setups (Figure 9g–i). For the flat rigid case accuracies were lower at 30.5 and 51.4 mmHg, but still above 90%. The tendency was to underestimate the pressure. Repeatability of between 1.1–3.5%, with one outlier of 5.5%, was achieved. The average accuracies of dynamic pressure readings, including repeatability in the form of absolute deviations, are summarised in Table 3.

**Figure 10:**
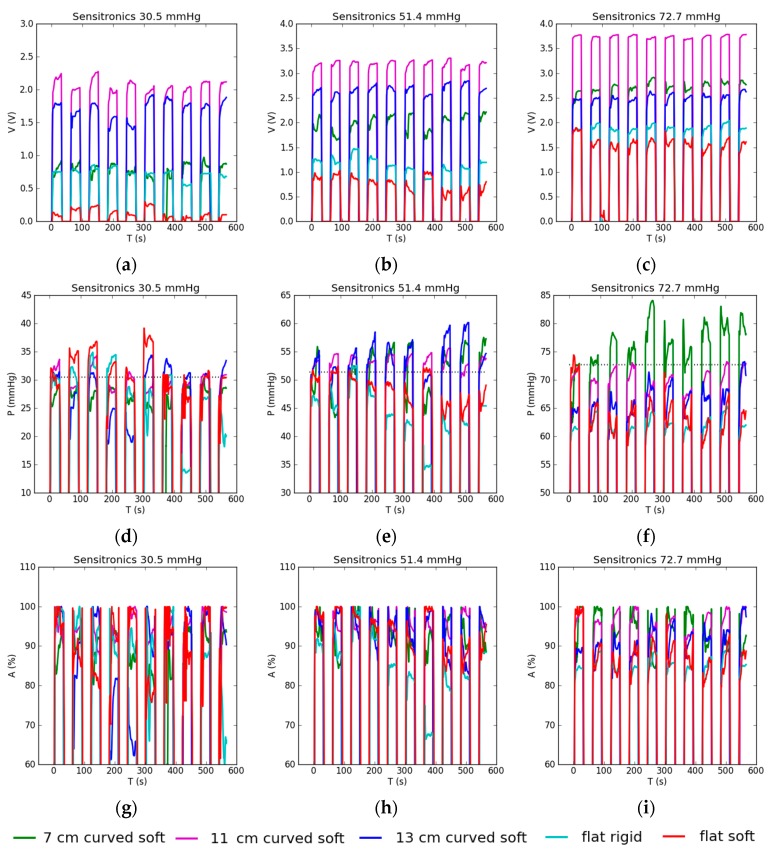
Dynamic testing results for Sensitronics sensor with applied pressure shown in the title: (**a**–**c**) sensor output voltage reading; (**d**–**f**) sensor pressure reading when sensor is recalibrated before each test. Dotted lines show corresponding applied gold standard pressures; (**g**–**i**) accuracy of the sensor pressure reading.

Dynamic testing using the Sensitronics sensor produced large differences in output voltages for different test setups, consistent with its static behaviour (Figure 10a–c); curved surfaces resulted in higher output voltage than flat surfaces. The 11 cm curved surface produced the highest voltage, despite not having the largest or smallest radius of curvature. Both upward and downward output voltage drifts were present through the cycle, as well as mid-cycle changes of drift direction. Output was visibly less smooth than for the Peratech sensor. Somewhat erratic behaviour was recorded on all type of surfaces, suggesting that it originates from the sensor construction.

Again, after individual calibration post-processing, reasonably consistent pressure readings were obtained (Figure 10d–f). Measured pressure readings agreed well with applied pressures of 30.5 and 51.4 mmHg. At 51.4 mmHg, curved setups overestimated the pressure and flat setups underestimated it. At 72.7 mmHg, in most setups pressure was underestimated, except for the 7 cm curved soft case, in which pressure was overestimated. Erratic drift tendencies within the cycle carried over into pressure outputs.

The Sensitronics sensor has lower accuracy (Figure 10g–i) than the Peratech sensor, starting from 84.2% for the flat rigid case, around 90% at 30.5 mmHg for the rest of the cases, and reaching 94.3% for one medium and one high pressure case. Overall repeatability was lower for this sensor as well, ranging from 1.2 to 7.2% with one outlier at 11.2%. The flat rigid case turned out to be the least accurate and 11 cm curved the most accurate. Results are summarised in Table 3.

## 4. Discussion

Our evaluation of the influence of the stiffness and curvature of surface on output voltage results using both sensors showed a consistent pattern in which voltage correlates to the type of underlying surface. The surface type affected the Sensitronics sensor more powerfully, as reflected in large differences in sensor output voltages between the setups; they also caused significant shifts in calibration curves. More drift was observed over time at low applied pressure. Flat setups resulted in lower voltages than curved setups across all pressure levels for the Sensitronics sensor. This sequence, however, was not observed with the Peratech sensor, which produced lower output voltages on flat surfaces and higher voltages at curved surfaces at high pressure, but had higher output voltage on flat surfaces and lower voltage on curved surfaces and occasionally mixed and overlapping results at lower applied pressures. The Peratech sensor displayed very stable behaviour, with less drift than the Sensitronics sensor; the surface type has minimal influence on its performance attributes, making it suitable for long-term measurements on both flat and curved surfaces with various radii of curvature. As the voltage graphs often overlap for the Peratech sensor, it can be concluded that the effect of different surfaces underneath the sensor are not distinguishable from inevitable variations that occur in repeated measurement.

Applying a linear fit for flat rigid calibration to the output voltage data, as would be the case if the curvature was ignored, resulted in substantial deviations of pressure readings depending on the underlying surface and sensor. This variation was insignificant for the Peratech sensor but significant (indicating twice or more the applied pressure) for the Sensitronics sensor. These findings show that the underlying surface affects sensors to different extents, and underline the need to apply different calibrations depending on the surface under the Sensitronics sensor.

Once the pressure calibration was applied individually for each surface type, the measured values of pressure were very close to each other, with frequent overlapping—an intended outcome of the calibration process. High accuracy was recorded at medium and high pressure levels. Repeat tests resulted in slightly different output values in dynamic tests. Both sensors had acceptable repeatability, with the Peratech sensor giving more consistent results than the Sensitronics device. Further study is needed in order to draw statistically valid conclusions on behaviour under static pressure application.

Manufacturers designed, calibrated and tested these sensors on flat rigid surfaces [26,27]. Logic dictates that soft substrates could affect the pressure distribution within the sensor. A curved substrate introduces additional complexity due to bending of the sensor and possible gaps between the sensor and the t-pin. Ideally, the t-pin base should follow the radius of curvature of the substrate for good fit. However, it is presumed that the sensor sinks into a soft substrate material, thus reducing bending and improving contact with the t-pin. This effect will reduce progressively for smaller radii of curvature. Further studies are suggested to determine the radius of curvature at which sensor output accuracy becomes unacceptable. Flat surfaces produced highly accurate results on multiple occasions; however, so did some of the curved surfaces, after appropriate calibration was applied. While the sensors behaved differently, no unified pattern of dependence on the radius of the surface curvature could be determined. This suggests that complex forces come into play when thin film sensors are used on soft curved surfaces; controlled experiments are needed to characterise these forces more accurately. The internal construction of the sensor and its response to bending seems likely to play an important role as well.

Counterintuitively, the flat rigid test setup was neither the most accurate nor exhibited the highest or lowest parameter values. In addition, the increase in output voltage did not follow the increase in curvature from flat to smallest radius for either sensor, except in the case of the Peratech sensor under low and medium pressures. The Sensitronics sensor showed a tendency to produce higher output voltage with curved test surfaces than flat surfaces. For dynamic tests, the stiffness of the substrate probably affected the sensor performance due to delayed response. However, we found no disadvantage in using a soft substrate. Other effects of surface characteristics such as, for example, roughness, could be investigated in future research.

Force-sensing resistors exhibit some degree of hysteresis and can return up to +/−5% different readings when slowly adding and slowly removing pressure, which should be considered for relevant applications. However, our study focused mainly on applications such as pressure garments in which the loading and unloading of pressure is not critical and will occur mainly due to the patient walking.

## 5. Conclusions

We evaluated the influence of the substrate stiffness and curvature using two commercial flexible resistive pressure sensors, made by Peratech and Sensitronics, on flat and curved surfaces. The sensors were selected from a previous study of a wider range of sensors due to their high accuracy and consistency. Flat rigid, flat soft silicone and three cylindrical silicone surfaces of radii of curvature of 3.5 cm, 5.5 cm and 6.5 cm were used as substrates under the sensors. Pressures of 30.5, 51.4 and 72.7 mmHg were applied via standard weights on a t-pin. For static testing, sensors were under continuous pressure for eight hours. For dynamic testing, 10 cycles of pressure application of 30 s duration were evaluated. The Peratech sensor was found to average 94% accuracy for both static and dynamic measurements on all the substrates, with accuracies across test setups ranging from 83.7% to 98.7%. The Sensitronics sensor was less accurate, with average accuracy of 88% and a range of 75.5–96.4%. The Sensitronics sensor displayed large variations and the Peratech sensor displayed moderate variations in output voltage depending on the underlying test surface, which resulted in corresponding variations in the output pressure readings of the sensor compared to the actual applied pressure value if flat rigid calibration was used for all surfaces. These variations were counteracted by individual pressure calibration for each type of surface, which operated as intended to reduce the deviation of measured pressure from that actually applied. At medium and high pressure levels of 51.4 and 72.7 mmHg, pressure readings were more accurate than at 30.5 mmHg. This study confirms that the output of the sensors changes depending on the underlying surface, but sensors are affected to a different extent depending on their construction. Therefore, some sensors, like the Sensitronics model tested here, require individual surface calibrations and would be very inaccurate without them. Other sensors, like the Peratech QTC^TM^ SP 200-10, tolerate different surfaces significantly better and could be used with a single calibration with acceptable accuracy and thus reduce the amount of initial setup required before measuring. This is an important consideration when choosing a sensor for a pressure-sensing device and its calibration method for many health care applications.

## Figures and Tables

**Table 1:** Geometry and sensing properties of sensors used for evaluation.

Sensor	Manufacturer	Sensing Area	Claimed Operating Range
QTC^TM^ SP 200-10	Peratech Ltd.	10.0 mm	0.1–20 N [26]
Half Inch ThruMode FSR	Sensitronics^®^ Inc.	12.7 mm	0.3–30 psi [27]

**Table 2:** Average accuracy in static pressure measurements.

Sensor	Surface Type	Accuracy, %		
		30.5 mmHg	51.4 mmHg	72.7 mmHg
Peratech	Flat rigid	90.0	91.7	98.7
	Flat soft	98.0	97.2	96.5
	7 cm curved soft	94.1	92.1	91.5
	11 cm curved soft	95.8	95.4	91.9
	13 cm curved soft	83.7	98.2	95.6
Sensitronics	Flat rigid	86.8	96.4	80.6
	Flat soft	-	94.6	75.5
	7 cm curved soft	89.7	89.8	90.0
	11 cm curved soft	91.2	92.8	92.4
	13 cm curved soft	84.2	87.1	85.0

**Table 3:** Average accuracy in dynamic pressure measurements.

Sensor	Surface Type	Accuracy, %		
		30.5 mmHg	51.4 mmHg	72.7 mmHg
Peratech	Flat rigid	90.8 ± 5.5	91.9 ± 3.5	96.0 ± 1.7
	Flat soft	97.9 ± 1.2	95.1 ± 3.0	97.1 ± 1.1
	7 cm curved soft	95.6 ± 2.9	96.6 ± 2.7	97.5 ± 1.6
	11 cm curved soft	97.0 ± 2.4	95.3 ± 2.3	97.8 ± 1.4
	13 cm curved soft	94.3 ± 1.7	95.9 ± 2.3	96.1 ± 1.8
Sensitronics	Flat rigid	84.2 ± 11.2	84.7 ± 6.2	85.3 ± 2.0
	Flat soft	90.6 ± 6.1	94.2 ± 3.1	90.1 ± 2.7
	7 cm curved soft	90.9 ± 3.2	92.4 ± 3.4	94.3 ± 2.6
	11 cm curved soft	95.1 ± 2.8	94.6 ± 1.2	97.9 ± 1.7
	13 cm curved soft	90.5 ± 7.2	91.8 ± 4.9	93.0 ± 2.2
